# Use of Traction Table for Reducing Complex Distal Femur Fractures: A Technical Trick

**DOI:** 10.7759/cureus.23889

**Published:** 2022-04-06

**Authors:** Joshua Rui Yen Wong, Stamatios Tsamados, Akash Patel, Parag Jaiswal

**Affiliations:** 1 Trauma and Orthopaedics, University College Hospital, London, GBR; 2 Trauma and Orthopaedics, Royal Free Hospital, London, GBR

**Keywords:** open reduction internal fixation, intra-articular fracture, complex distal femur fracture, orif, minimally invasive plate osteosynthesis

## Abstract

Complex intra-articular distal femur fractures (AO 3.3.C) present many challenges, especially with regard to patient preparation and approach. Traditionally, patients lie supine with a bolster or triangular wedge placed below the knee to allow knee flexion. This corrects the hyperextension deformity of the distal femoral fragment caused by the gastrocsoleus complex. Although well established, the minimally invasive plate osteosynthesis (MIPO) technique presents with limitations, especially with regard to patient preparation and approach. In this case report, we describe a lateral approach to the MIPO technique using the Eschmann traction table with the tibia nailing attachment and femoral condyle pads to achieve and maintain satisfactory fracture reduction throughout the procedure. Importantly, this also allows less dependency on surgical assistance. To our knowledge, this is the first case report describing a lateral approach to the MIPO technique using the Eschmann traction table for the reduction of complex distal femur fractures.

## Introduction

Any fracture extending from the distal metaphyseal-diaphyseal junction of the femur to the articular surface of the femoral condyles is classified as a distal femur fracture. The estimated frequency of distal femur fractures is approximately 3% of all femoral fractures [1]. Although uncommon, distal femur fractures are severe and require complex surgical management. Conventionally, open reduction and internal plate fixation of distal femoral fractures are fraught with problems. For instance, fixation with classic intramedullary osteosynthesis was found to be unstable [2], whereas open reduction and internal fixation by classic plates would require a sizeable incision with important deperiostation. Given the potential complications associated with these conventional techniques, the concept of indirect reduction was developed. Krettek et al. reported several minimally invasive plate osteosynthesis (MIPO) techniques for distal femoral fractures [3]. The MIPO techniques allow for the fracture site to be unexposed while transforming the conventional plate as an internal extramedullary splint independent of compression or lag screw application [3]. As such, MIPO techniques are now well established and indicated in all complex long bones fractures that are unsuitable for intramedullary osteosynthesis [4]. Despite this, MIPO techniques are not perfect and present with limitations, especially with regard to patient preparation and approach. In this case report, we describe an alternative approach to the MIPO technique.

## Case presentation

**Figure 1:**
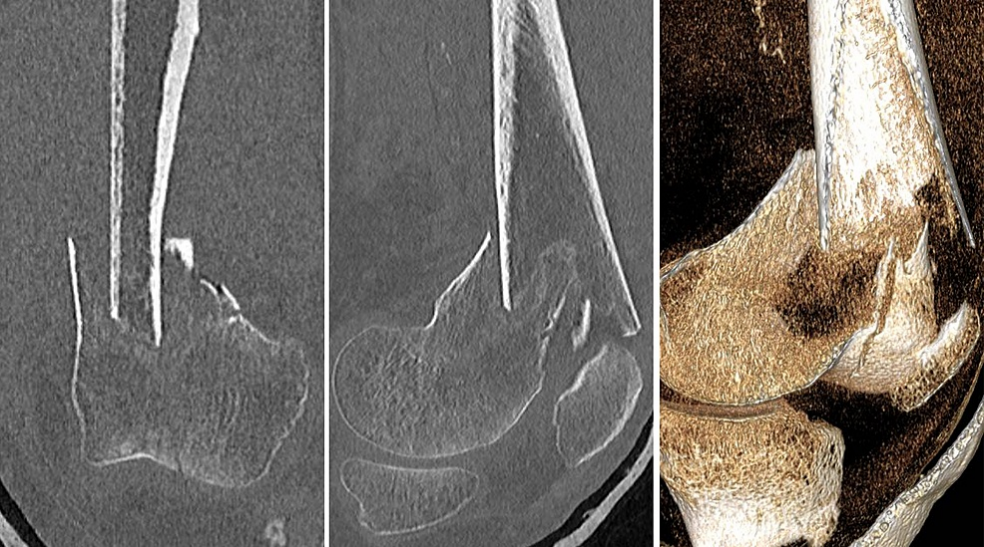
CT scans for preoperative surgical planning. Coronal (left), sagittal (middle), and 3D reconstruction (right) views displaying the intra-articular distal femur fracture. CT: computed tomography; 3D: three-dimensional

An 82-year-old lady presented to the emergency department after she injured her left leg following a mechanical fall down a flight of stairs. She was initially assessed using the principles of the Advanced Trauma Life Support program. After the primary and secondary surveys, she was diagnosed with an isolated, closed intra-articular fracture of the left distal femur. As shown in Figure 1, plain radiographs and computed tomography scan of the left knee demonstrated an AO 3.3.C.2 fracture [5].

Surgical technique

**Figure 2:**
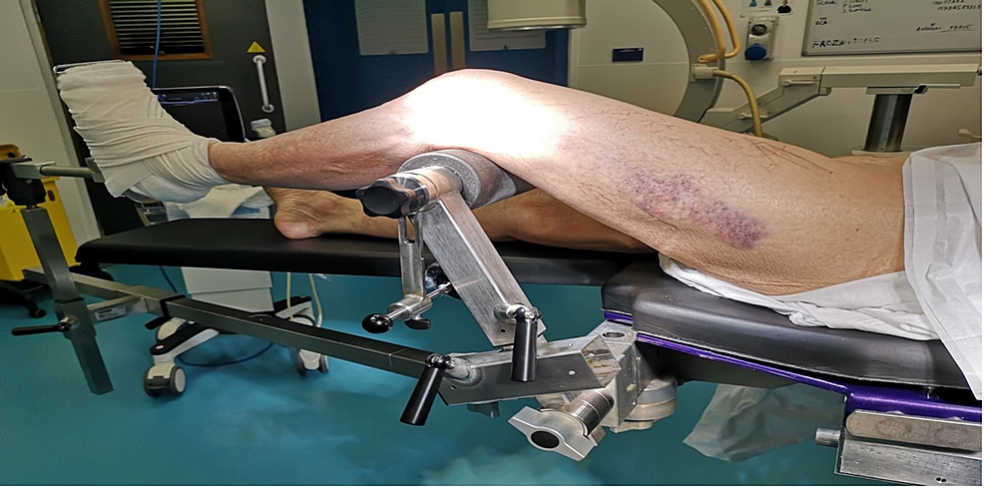
Lateral view of patient positioning set up with the tibial nailing attachment with femoral condyle pads.

**Figure 3:**
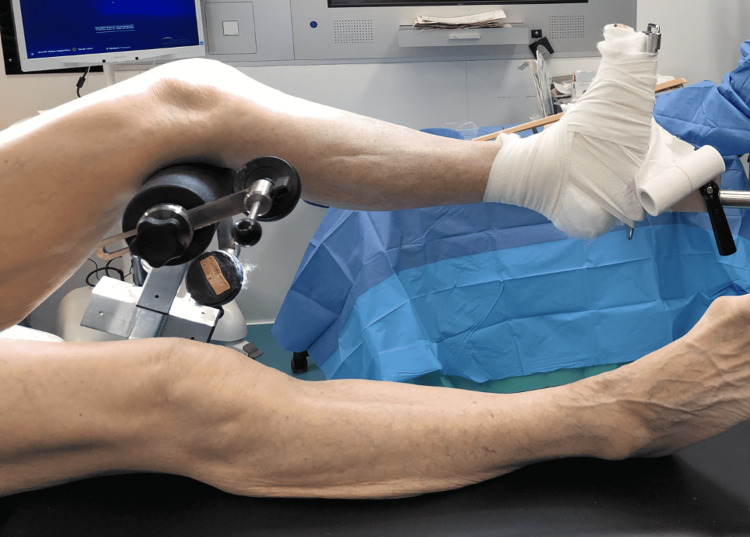
Medial view of the patient positioning set up with the tibia nailing attachment with femoral condyle pads.

**Figure 4:**
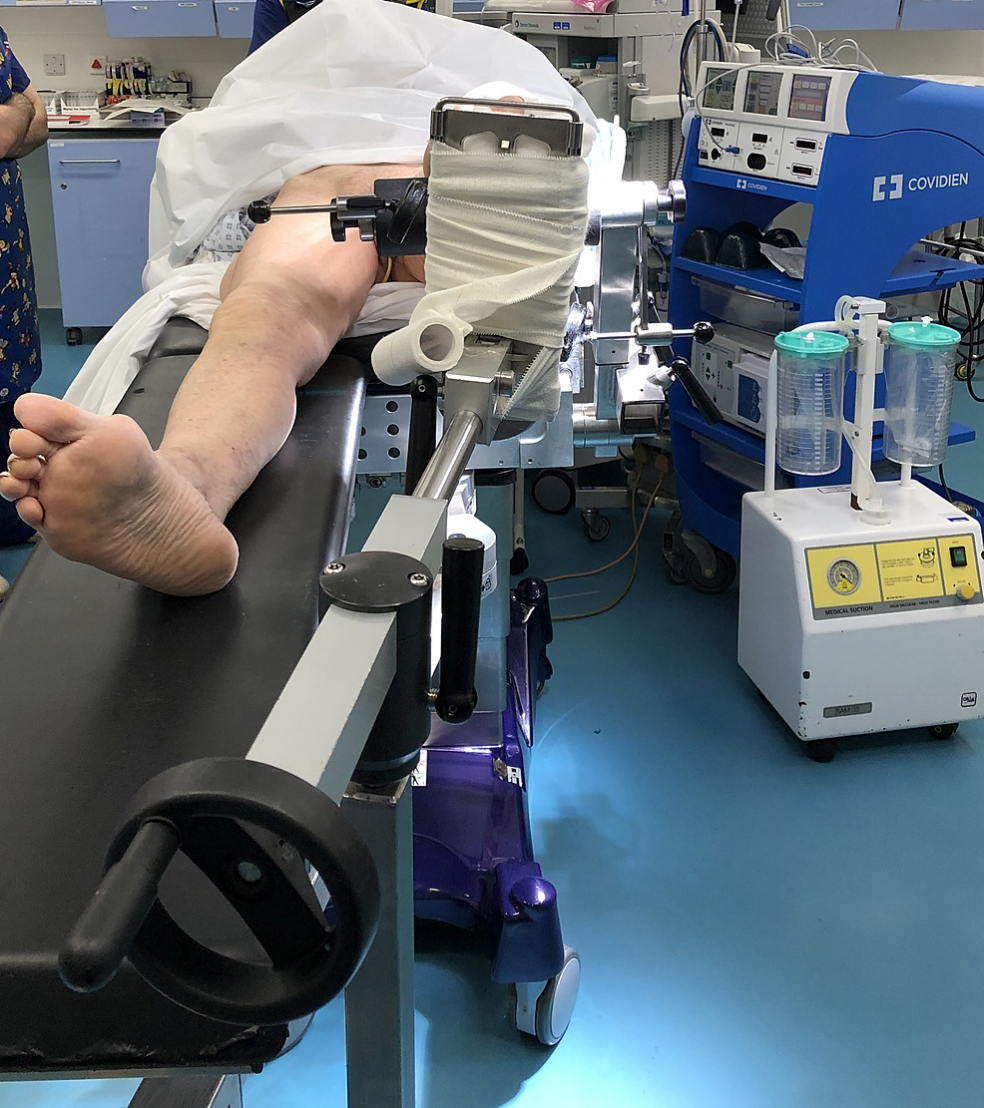
Distal view showing the right leg in a neutral supine position with the preoperative leg support.

In this indirect reduction technique, the patient, under general anaesthesia, was placed in a supine position on a radiolucent Eschmann traction table (Eschmann Equipment, Peter Road, Lancing, England). For the left leg, the T30 orthopaedic trunk hip section was replaced with a tibia nailing attachment under the distal femoral fragment and femoral condyle pads to support medial and lateral tibial condyles, as shown in Figure 2. The left foot was wrapped in wool and secured to the traction unit footplate. An adhesive bandage was used to secure the position of the foot, as shown in Figure 3. The right leg was left in a neutral supine position with the leg support, as shown in Figure 4.

**Figure 5:**
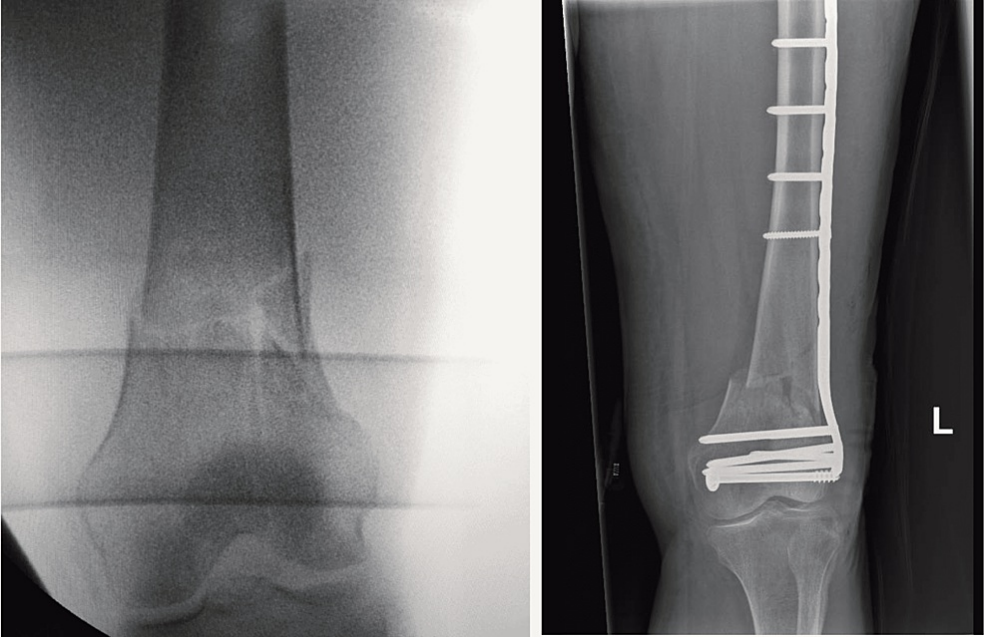
Intraoperative radiographs with the described setup taken by the image intensifier showing before (left) and after fixation (right).

An image intensifier was used to ensure good reduction was achieved preoperatively. Preoperative reduction manoeuvres were performed to maintain a satisfactory reduction in coronal and sagittal planes under image intensifier guidance, as shown in Figure 5.

Following a satisfactory reduction of the fracture and allowing sufficient space for the C-arm to obtain intraoperative radiographs, a solution of chlorhexidine 2% was used for skin antisepsis. The operative field was draped with adhesive drapes and iodophor antimicrobial surgical incise drapes. The procedure was completed without complications using the lateral approach and a variable angle condylar locking plate using the aiming arm for percutaneous screw insertion. It should be noted that the tibia nailing attachment was applied just proximal to the popliteal fossa to avoid pressure on the neurovascular structures and at the level of the fracture site to correct the flexion deformity of the distal fragment while reducing the fracture satisfactorily.

## Discussion

The current literature describes other techniques, such as the lateral decubitus approach. Limitations of this include difficulty in maintaining spinal precautions and obtaining intraoperative images [6]. However, as seen in this case report, the supine position of the technique reduces these problems.

Traditionally, patients lie supine, with a bolster or triangular wedge, placed below the knee to allow for knee flexion [7,8]. This position provides support for the knee and relaxes the pull of the gastrocsoleus muscle complex on the posterior femoral condyles. This reduces hyperextension deformity of the distal fragment. However, this traditional technique requires another assistant to maintain femoral reduction and application of traction during internal fixation. Given that a surgical assistant may not always be available, the aforementioned technique not only allows for the operating surgeon to perform it individually but also retains the traditional position with knee flexion without obstructing the intraoperative imaging. Additionally, the height of the tibial nailing attachment can be adjusted to improve the fracture reduction in the sagittal plane, along with the position of the lower limb in the traction table (adduction/abduction) which can further improve reduction in the coronal plane.

## Conclusions

To our knowledge, a lateral approach of the MIPO technique for the reduction of distal femur fractures has not been previously described in the literature. This approach has been convenient to maintain satisfactory fracture reduction throughout the whole procedure. We suggest this way of lower limb positioning not only to facilitate optimal reduction in complex distal femur fractures but also to reduce dependency on surgical assistance for the MIPO technique.
